# Distinct genetic requirements underlie LITE-1-dependent photosensation and avoidance of high concentration of diacetyl in *C. elegans*

**DOI:** 10.64898/2026.10.13.758841

**Published:** 2026-10-14

**Authors:** Alan Koh, Changchun Chen, André EX Brown

## Abstract

The eyeless nematode *C. elegans* can detect and respond to aversive blue and ultraviolet light using the gustatory receptor homolog LITE-1. In addition to its role in phototransduction, LITE-1 also functions as a chemoreceptor for the odorants diacetyl and 2,3-pentanedione. However, whether these different sensory modalities rely on the same downstream signalling components is unknown. In this study, we provide behavioural evidence that unlike its LITE-1-dependent photosensory function which relies on cGMP signalling components, its chemosensory function remains intact in mutants with disrupted cGMP signalling.

